# The impact of meteorological conditions on cardiovascular and cerebrovascular diseases in different microclimatic zones of low-latitude mountainous areas

**DOI:** 10.3389/fpubh.2024.1447910

**Published:** 2024-12-06

**Authors:** Zhengjing Du, Fang Xiong, Yanjing Tang, Xiaoling Xia, Yuandong Hu, Fangfang Wang

**Affiliations:** ^1^Guizhou Mountainous Meteorological Science Research Institute, Guiyang, Guizhou, China; ^2^Guizhou Meteorological Data Center, Guiyang, Guizhou, China; ^3^Guizhou Meteorological Service Center, Guiyang, Guizhou, China; ^4^Guizhou Center for Disease Control and Prevention, Guiyang, Guizhou, China

**Keywords:** meteorological conditions, microclimate, cardiovascular and cerebrovascular diseases, distributed lag non-linear model, temperature variation

## Abstract

**Introduction:**

Epidemiological evidence suggests cerebrovascular disease (CVD) incidence is correlated to meteorological conditions. However, research on the primary meteorological factors influencing the incidence of CVD and their influence thresholds in low-latitude mountainous regions remains insufficient. We aimed to investigate the association between meteorological conditions and CVD occurrence in Guizhou province.

**Methods:**

Utilizing daily incidence cases for CVD alongside concurrent meteorological data from four microclimate representative stations in Guizhou Province during 2021-2022, we firstly performed random forest and decision tree analysis to identify the significant meteorological factors influencing the incidence of CVD, and then we applied distributed lag non-linear model (DLNM) to assess the effect of meteorological factors on CVD incidence.

**Results:**

Critical meteorological factors associated with CVD incidence include diurnal temperature range (DTR), temperature change between neighboring days (TCN), diurnal pressure range (DPR), pressure change between neighboring days (PCN), and minimum temperature. The risk of CVD incidence markedly increased with narrow DTR, positive TCN, and colder conditions. The thresholds of minimum temperature and TCN droped with the decrease of annual average temperature in microclimate zone. In the middle subtropical region of Jiangkou, the northern subtropical region of Xishui, and the warm temperate region of Shuicheng, there was a risk of CVD when the minimum temperature falled below 12.2°C, 10.7°C, and 6.7°C, respectively. When TCN exceeded the critical threshold (0.2°C in Shuicheng, 0.3°C in Xishui, and 0.4°C in Jiangkou and Ceheng), the risk of CVD incidence increased linearly. DPR and PCN displayed varied thresholds across different microclimate without consistent patterns. DPR in middle subtropical region of Jiangkou and warm temperate region of Shuicheng played a protective role, while the risk of the disease increased linearly when DPR was lower than 3.2 hPa and 2.7 hPa in south subtropical region of Ceheng and north subtropical region of Xishui, respectively. The short-term effects within 5 days with small DTR and narrow positive TCN were more obvious. The thresholds and lag times of other meteorological environmental factors remained indistinct in the four microclimate zones.

**Discussion:**

Our findings delineated the common characteristics of the effect on the incidence of CVD of meteorological environments in various microclimate zones, and clarified the influence of minimum temperature and TCN exhibited spatial heterogeneity, Which may be assistance for meteorological risk forecasting in CVD prevention and control.

## 1 Introduction

The influence of meteorological factors on the risk of CVD incidence is biologically plausible and supported by epidemiological evidence (1, 2). With global climate warming the impact of meteorological conditions on CVD incidence or mortality has been extensively investigated. Exposure to extreme temperatures, both high and low, increases the risk of CVD mortality (3–5). Studies in Brazil and China have demonstrated (6–9) that extreme temperatures, daily temperature fluctuations, and 24-h temperature variations are associated with heightened risks of CVD mortality or hospitalization. However, regional variations in temperature tolerance among populations lead to differing temperature thresholds for CVD risks, showing spatial heterogeneity. Other factors such as atmospheric pressure, humidity, and wind also influence CVD incidence. For example, ischemic stroke in Tianjin is positively correlated with diurnal temperature range and atmospheric pressure (average and minimum values) (10). Mortality rates increase on days with significant temperature rises and pressures drops in Prague and the Czech Republic (11, 12). Katarzyna Zareba’s analysis of stroke patients in Katowice indicates that lower average temperatures, reduced sunshine, high humidity, and high wind speeds increase the risk of winter strokes (13). These findings underscore that abrupt meteorological changes can trigger CVD events.

Previous research on the impact of meteorological environments on CVD has predominantly focused on individual cities, lacking comparative studies across different climatic zones. China boasts a vast territory and a diverse array of climates, encompassing 12 temperature zones, 24 dry and wet regions, and 56 climatic zones across the nation (14). Located on the eastern edge of the Yunnan-Guizhou Plateau, Guizhou experiences distinct seasons, mild winters, cool summers, abundant rainfall, frequent cloudy days, high humidity, and numerous rainy days. Guizhou’s climate combines elements of subtropical plateau monsoon climate with distinct mountainous three-dimensional climate characteristics. It can be divided into four microclimate zones (15): South subtropical, Central subtropical, North subtropical, and Warm temperate zones. CVD ranks among the top three causes of death in Guizhou Province. Given that CVD ranks among the top three diseases with the highest mortality rates in Guizhou (16) and has a profound impact, it is crucial to explore the impact of meteorological environmental factors on CVD in this low-latitude mountainous area.

The relevant meteorological factors include temperature, atmospheric pressure, humidity, and wind. Identifying key factors influencing CVD incidence and understanding their spatial heterogeneity necessitates thorough exploration and clarification. Consequently, this study selects four representative stations across Guizhou’s diverse Micro-climatic zones to examine CVD morbidity and meteorological conditions. This analysis aims to identify principal factors affecting CVD morbidity in Guizhou and their impacts. Anticipated outcomes include the development of targeted CVD morbidity forecasting services using weather forecast products and the formulation of effective CVD risk prevention and control strategies.

## 2 Materials and methods

### 2.1 Materials

This study employs daily CVD incidence data from four microclimate representative stations (Ceheng in the South Subtropical Zone, Jiangkou in the Central Subtropical Zone, Xishui in the North Subtropical Zone, and Shuicheng in the Warm Temperate Zone) collected by the Guizhou Center for Disease Control and Prevention for the years 2021 to 2022. Concurrent meteorological data, including temperature, rainfall, atmospheric pressure, humidity, wind, and sunshine, were obtained from the Guizhou Meteorological Data Center. The CVD data encompass variables such as gender, age, place of residence, disease diagnosis type (ICD-10 codes), and date of onset. Meteorological elements include daily average temperature, maximum temperature, minimum temperature, daily rainfall, daily average pressure, daily average humidity, daily average wind speed, and sunshine duration. Derived variables include diurnal temperature range(DTR), temperature change between neighboring days (TCN) (17), consecutive rainy days (sequential days with rainfall exceeding 0.1 mm), consecutive dry days (sequential days without rainfall), diurnal pressure range (DPR), and pressure change between neighboring days (PCN).

### 2.2 Quality control

The CVD and meteorological data underwent verification by the respective disease control and meteorological departments to confirm data integrity. The meteorological datasets were free of duplicates and missing values, and critical identifiers (patient ID) in the disease datasets were screened for duplicates. The cumulative number of valid cases for Ceheng, Jiangkou, Xishui, and Shuicheng totaled 2,286, 2,451, 7,682, and 5,328, respectively. All data were verified to be free from outliers or logical inconsistencies.

### 2.3 Methods

A Poisson regression served as the link function to configure a distributed lag non-linear model (DLNM), described in Equation 1:


(1) $$g\left[E\left(Y_{t}\right)\right]=\alpha +\overset{L}{\underset{l=0}{\sum }}f•x_{t-1}w\left(1\right)+g\left(t\right)+\overset{K}{\underset{k=1}{\sum }}\gamma_{k}\mu_{k}$$

where *g* represents the family of link functions; *Y_t_* denotes the expected number of cases on day t; *α* is the intercept; *w(l)* is the lag-response function; *f·x_t-1_*is the exposure-response function; *f* is the basis function for the independent variable; *l* is the lag time; *L* is the maximum lag time, set at 30 days for this study (18); x denotes the exposure factors, specifically meteorological elements here; g*(t)* is a spline function managing the degrees of freedom for the time trend, with six degrees of freedom per year recommended for monitoring short-term acute effects such as emergency admissions and outpatient visits, as suggested by Fan et al. (19); *μ_k_* accounts for the influence of confounding factors, including meteorological and environmental elements (e.g., SO2, NO2, and PM10); and *γ_k_* denotes the parameters corresponding to these confounding factors. The choice of meteorological factors as confounding elements was based on minimizing the Akaike Information Criterion value (20), with degrees of freedom set to three (21, 22).

The impact of meteorological factors on CVD was adjusted for time trends, day-of-week effects, holiday effects, and air quality confounding factors, and expressed as relative risk (RR) and 95% confidence intervals (95% CI). A *p*-value of less than 0.05 was considered statistically significant.

To evaluate the significance of various meteorological factors on CVD incidence, we employed two comparative machine learning methods: the Random Forest Classifier and the Decision Tree Classifier from the Python machine learning toolkit.

## 3 Meteorological conditions and CVD characteristics

### 3.1 Climate conditions of the four representative stations

The average climate conditions of the four microclimate representative stations are presented in Table 1. The annual average temperatures at Ceheng, Jiangkou, Xishui, and Shuicheng are 19.5°C, 16.7°C, 13.7°C, and 12.9°C, respectively, with Ceheng being the warmest and Shuicheng the coolest. The average diurnal temperature range from 1991 to 2020 for these stations varies between 6.5°C and 8.6°C, with Xishui experiencing the narrowest range. The daily mean pressure range spans from 4.6 to 6 hPa, with Shuicheng, representing the warm temperate zone, having the smallest daily pressure range.

**Table 1:** Climate conditions and incidence of CVD in four microclimate representative stations.

Microclimate zone	Representative county	Average annual temperature (°C)	1991–2020 average DTR	1991–2020 average DPR	2021–2022 cumulative cases	2022 permanent resident population (10,000 people)
South Subtropical	Ceheng	19.5	8.6	6	2,286	18.85
Mid-Subtropical	Jiangkou	16.6	7.9	5.3	2,451	18.35
North Subtropical	Xishui	13.7	6.5	4.7	7,682	58.38
Warm Temperate	Shuicheng	12.9	7.9	4.6	5,328	62.26

### 3.2 Meteorological conditions and CVD characteristics

According to the CVD incidence data from the four representative stations (Table 2), Xishui recorded the highest number of cases, followed by Shuicheng, with Ceheng and Jiangkou reporting relatively fewer cases. Based on the permanent resident population in 2022, as indicated in Table 1, Jiangkou and Xishui have the highest average CVD incidence rates at 0.183 and 0.18 per 10,000 people, respectively. In contrast, Shuicheng has the lowest rate at 0.117 per 10,000 people, and Ceheng’s rate is 0.166 per 10,000 people. The peak incidence was on October 29, 2022, in Xishui, with 156 cases, all diagnosed as strokes.

**Table 2:** Description of CVD incidence and meteorological conditions in four microclimate representative stations from 2021 to 2022.

		Number of cases	Maximum temperature	Minimum temperature	Average humidity	Sunshine duration	Air velocity	DTR	TCN	DPR	PCN
On average	Ceheng	3.1	25.2	16.7	78.5	5.7	1	8.5	0	6.1	0
	Jiangkou	3.5	22.2	13.5	81.9	3.9	1.6	8.7	0	5.2	0
	Xishui	10.5	18.6	12.1	80.7	3	1.3	6.5	0	4.8	0
	Shuicheng	7.3	18.4	10.4	81.9	3.3	1.1	8	0	4.6	0
Minimal	Ceheng	0	6.1	2.4	45	0	0	1.4	-9	2.3	−9.6
	Jiangkou	0	−0.9	−3.1	34	0	0	0.8	−12.3	1.7	−10.4
	Xishui	0	−1.6	−5.8	36	0	0	0.7	−8.3	1.7	−11
	Shuicheng	0	−2.5	−5.1	22	0	0.1	0.9	−9.3	1.7	−8.9
25%	Ceheng	1	18.9	11.8	72	2	0.7	5.1	−1	4.8	−1.7
	Jiangkou	2	14	7	74	0	1.1	4.3	−0.9	3.9	−1.6
	Xishui	6	11.2	5.9	74	0	0.9	3.1	−0.8	3.5	−1.4
	Shuicheng	4	12.2	5	75	0	0.8	4.4	−1	3.5	−1.3
50%	Ceheng	3	26.8	17.5	78	6.6	0.9	8.5	0.3	5.9	−0.1
	Jiangkou	3	22.3	13.4	83	2.7	1.5	8.6	0.3	4.8	−0.2
	Xishui	10	18.8	12.1	82.5	0.9	1.2	6.1	0.2	4.3	−0.1
	Shuicheng	7	20.1	11.1	83	1.9	1.1	7.8	0.2	4.4	−0.1
75%	Ceheng	5	32.6	22.1	86	8.7	1.2	11.3	1	7	1.5
	Jiangkou	5	32.1	21.1	94	7.3	2	12.9	1.2	5.9	1.4
	Xishui	13	26.7	18.4	90	5.9	1.6	9.4	1.1	5.8	1.5
	Shuicheng	9	25.6	16.2	91	6.2	1.3	10.9	1.3	5.4	1.3
Greatest	Ceheng	17	38.6	26.2	100	12.2	3.7	20.7	6.9	17	13.2
	Jiangkou	14	41.6	26.7	100	12.5	5.4	22.9	6.1	16.6	13.2
	Xishui	156	36.5	25.3	100	12.8	4	18.7	5.2	15.1	11.5

Other meteorological elements show that the average humidity across the stations ranges from 78.5 to 81.9%. Jiangkou records the highest average wind speed at 1.6 m/s. The daily pressure range decreases with a decline in annual average temperature, and Xishui exhibits the narrowest diurnal temperature range at 6.5°C. On October 29, 2022, Xishui reported an average temperature of 11.8°C, a daily average pressure of 889.2 hPa, a wind speed of 1.4 m/s, and a humidity level of 87%, with no rainfall recorded that day.

## 4 The impact of meteorological conditions on CVD

### 4.1 Importance analysis of meteorological factors

Extensive research has demonstrated that factors such as temperature, pressure, humidity, wind, rainfall, and sunshine influence CVD incidence. However, these meteorological environmental factors constitute a composite environment, not isolated elements. In this complex setting, previous studies have been unable to fully assess the significance of these factors on CVD incidence. Consequently, this study examines the importance of meteorological environmental factors on CVD.

Utilizing Random Forest and Decision Tree models, we assessed the importance of meteorological environments on CVD incidence. Table 3 identifies factors with an importance contribution value exceeding 0.07 among the 14 meteorological elements. Notably, DTR, TCN, DPR, PCN, and minimum temperature emerge as the most impactful on CVD incidence. In the warm temperate zone, Shuicheng’s minimum temperature has a negligible impact on CVD. PCN proves to be non-significant for CVD in Ceheng and Shuicheng, according to the Decision Tree model. Variable importance of wind speed, minimum pressure, maximum temperature, and sunshine duration differs across climate zones, while daily average pressure and rainfall exert the least influence on CVD incidence. Consequently, this study will concentrate on analyzing the impact of the five significant factors, such as DTR, on CVD.

**Table 3:** List of importance contribution values of meteorological environmental factors in four microclimate zones under two models.

Factor	Ceheng		Jiangkou		Xishui		Shuicheng	
	Random forest	Decision tree	Random forest	Decision tree	Random forest	Decision tree	Random forest	Decision tree
DTR	0.071	0.082	0.074	0.092	0.072	0.085	0.075	0.081
TCN	0.076	0.074	0.08	0.086	0.073		0.074	0.074
DPR	0.07	0.081	0.077	0.088	0.074	0.08	0.072	
PCN	0.078		0.077	0.08	0.078	0.113	0.073	
Minimum temperature	0.073	0.07	0.07	0.072	0.07	0.078		
Wind speed			0.071	0.086		0.08		0.073
Minimum pressure		0.083		0.07	0.072			
Maximum temperature			0.071	0.076	0.07			
Sunshine duration	0.071	0.072						0.071
Maximum pressure			0.07					0.077
Relative humidity		0.076					0.071	
Average temperature					0.071			0.073

### 4.2 Diurnal temperature range

**Figure 1:**
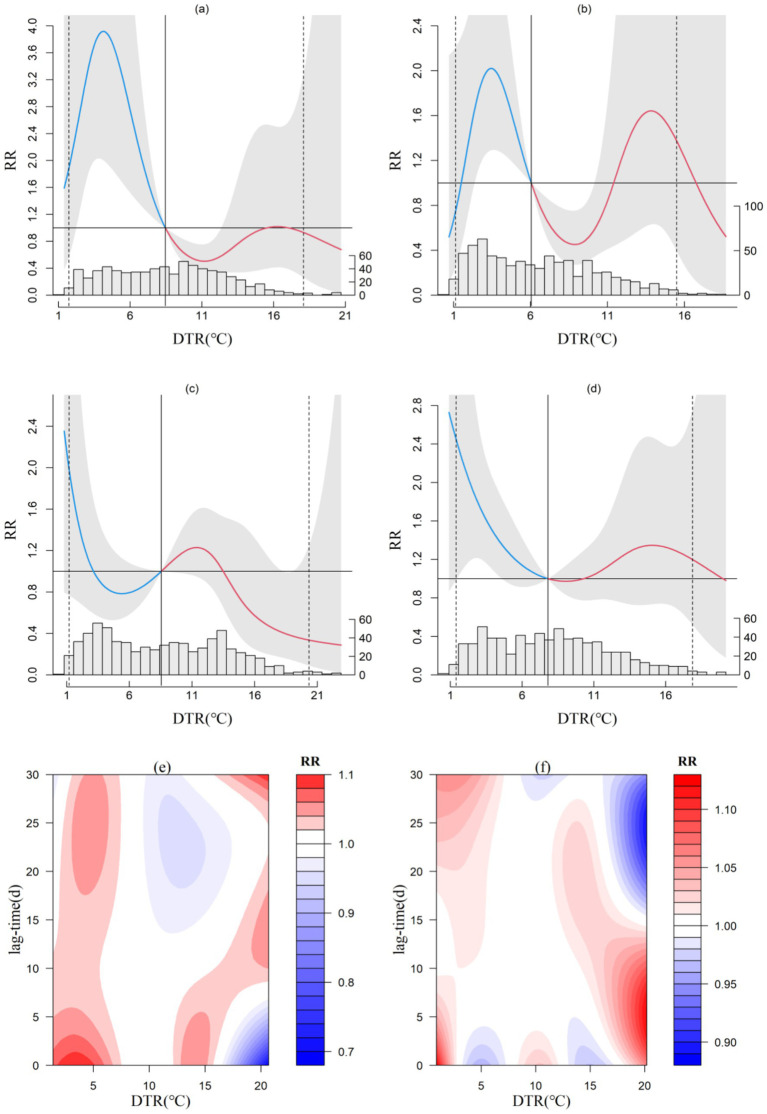
Exposure-response relationship between diurnal temperature range and CVD incidence in Ceheng (a), Xishui (b),Jiangkou (c) and Shuicheng (d) and lag effect diagram in Ceheng (e) and Shuicheng (f; the gray area in a, b, c, and d represents the 95% confidence interval for relative risk).

Relevant environmental biological studies (23) show that large DTR will affect humoral and cellular immune function, which may change autonomic nervous function and induce a series of CVD. The exposure-response relationship between the DTR and CVD incidence at the four microclimate representative stations is depicted. Ceheng (Figure 1a) and Xishui (Figure 1b) display an irregular “M”-shaped relative risk curve, while Jiangkou (Figure 1c) and Shuicheng (Figure 1d) exhibit an inverted “N”-shaped curve. The optimal DTR for minimizing CVD impact at Ceheng, Jiangkou, Xishui, and Shuicheng are 11.2°C, 22.9°C, 8.9°C, and 9.1°C, respectively, with Jiangkou also showing a critical point at 5.4°C. A consistent observation across the stations is that a narrower DTR correlates with a higher risk of CVD incidence. Specifically, at Ceheng, the risk of CVD is elevated when the DTR is between 1.4°C and 8.4°C. The other stations, Jiangkou, Xishui, and Shuicheng, show two high-risk intervals for CVD incidence: 0.8–3°C (8.7–13.4°C), 1.6–6°C (11.5–16.7°C), and 0.9–7.7°C (10.4–19.9°C), respectively, with the former intervals having higher relative risk (RR) values for DTR than the latter. In these regions, the RR increases almost linearly with each 1°C decrease in DTR, with increases of 0.58 and 0.25 at Jiangkou and Shuicheng, respectively. When DTR exceeds 8.5°C, 13.5°C or 16.8°C at Ceheng, Jiangkou and Xishui respectively, the RR value is less than 1, which may play a protective role.

Concerning the lag effect of DTR, both Ceheng (Figure 1e) and Shuicheng (Figure 1f) show notable short-term and medium-to-long-term effects with narrow ranges. At Ceheng, when DTR is below 7°C, elevated RR values are observed within a 5-day lag and between 17 to 30 days. At Shuicheng, a significant lag effect is evident within 5 days when DTR is below 2.5°C, and a round 17°C, significant effects manifest within 15 days, with the highest RR reaching 1.3. In the Central and North Subtropical zones (not shown), the lag effect mainly presents as short-term effects (within 5 days) when DTR is below 5°C.

### 4.3 Temperature change between neighboring days

**Figure 2:**
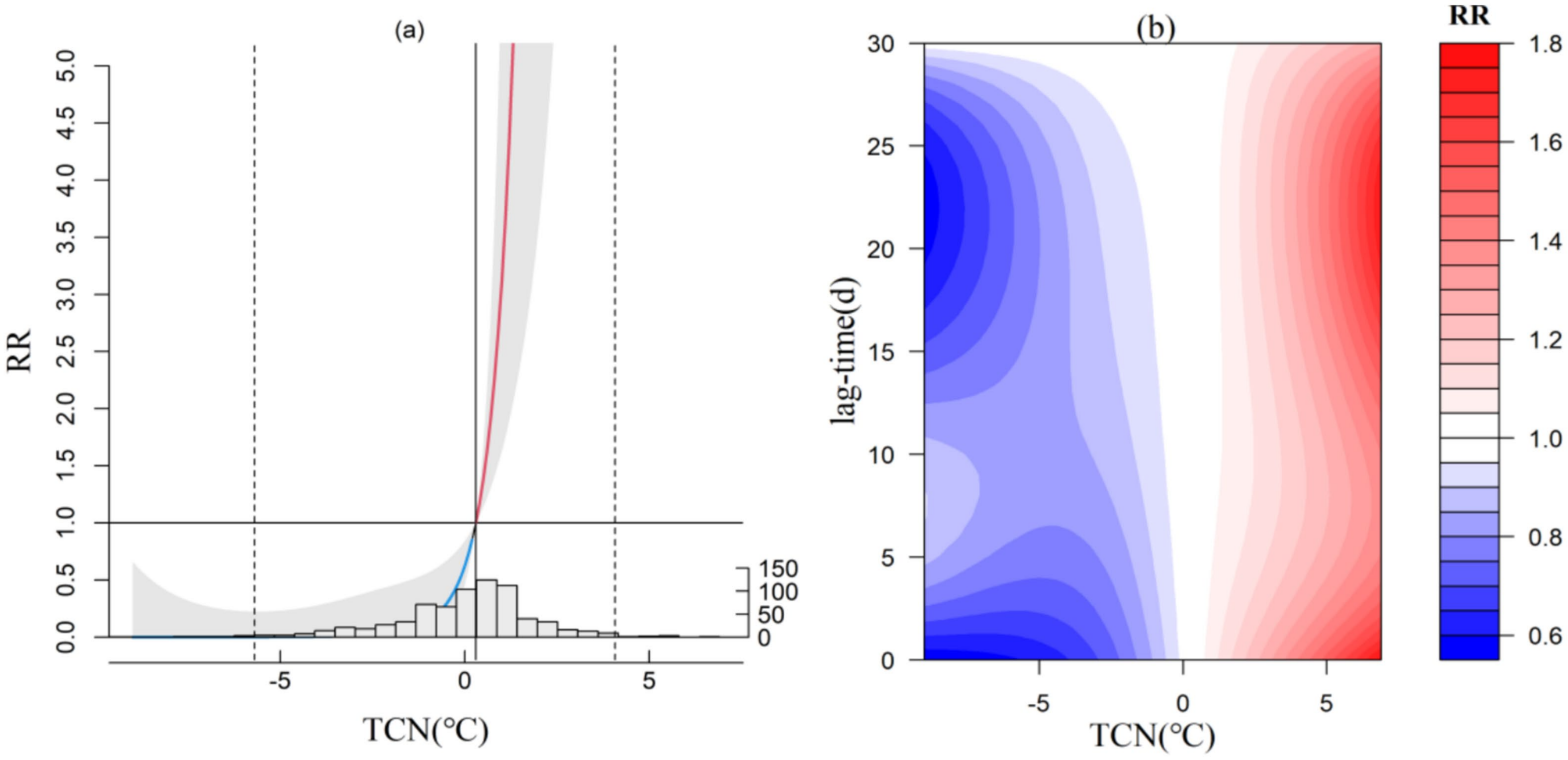
Exposure-response relationship between TCN and CVD incidence in Ceheng (a) and lag effect diagram (b; the gray area in a represents the 95% confidence interval for relative risk).

Temperature Change between Neighboring days (TCN) signifies periods of temperature decline or rise. Related epidemiological studies (24) suggest that when the TCN increases significantly, the human thermoregulatory system may struggle to adapt, leading to an increase in heart rate and blood pressure, a decrease in immune function, and potentially triggering circulatory system diseases. The exposure-response relationship between TCN and CVD incidence is consistent across the four microclimate zones. Using Ceheng as an example (Figure 2), a decrease in temperature is detrimental for CVD patients, which means a decrease in temperature may serve a protective function, while an increase in average daily temperature uniformly elevates the risk of CVD incidence. In the four microclimate zones, as the local annual average temperature rises, the critical threshold for TCN causing CVD incidence also increases slightly. When TCN exceeds the critical threshold (0.2°C in Shuicheng, 0.3°C in Xishui, and 0.4°C in Jiangkou and Ceheng), the risk of CVD incidence increases linearly.

Regarding the lag effect of TCN, both short-term and medium-to-long-term effects are significant at the South Subtropical representative station Ceheng and the warm temperate representative station Shuicheng when TCN increase is minimal. At Ceheng, with the next day’s temperature increase below 7°C, high RR values are noted within a 5-day lag and between 17 and 30 days. At Shuicheng, with the next day’s temperature increase (below 2.5°C) stably, a notable lag effect occurs within 5 days, and when the increase reaches 17°C, significant effects are observed within 15 days, with the highest RR peaking at 1.3. In the Central (not shown) and North Subtropical regions (not shown), the lag effect primarily manifests in short-term effects (within 5 days) when the next day’s temperature increase is below 5°C.

### 4.4 Daily pressure range

**Figure 3:**
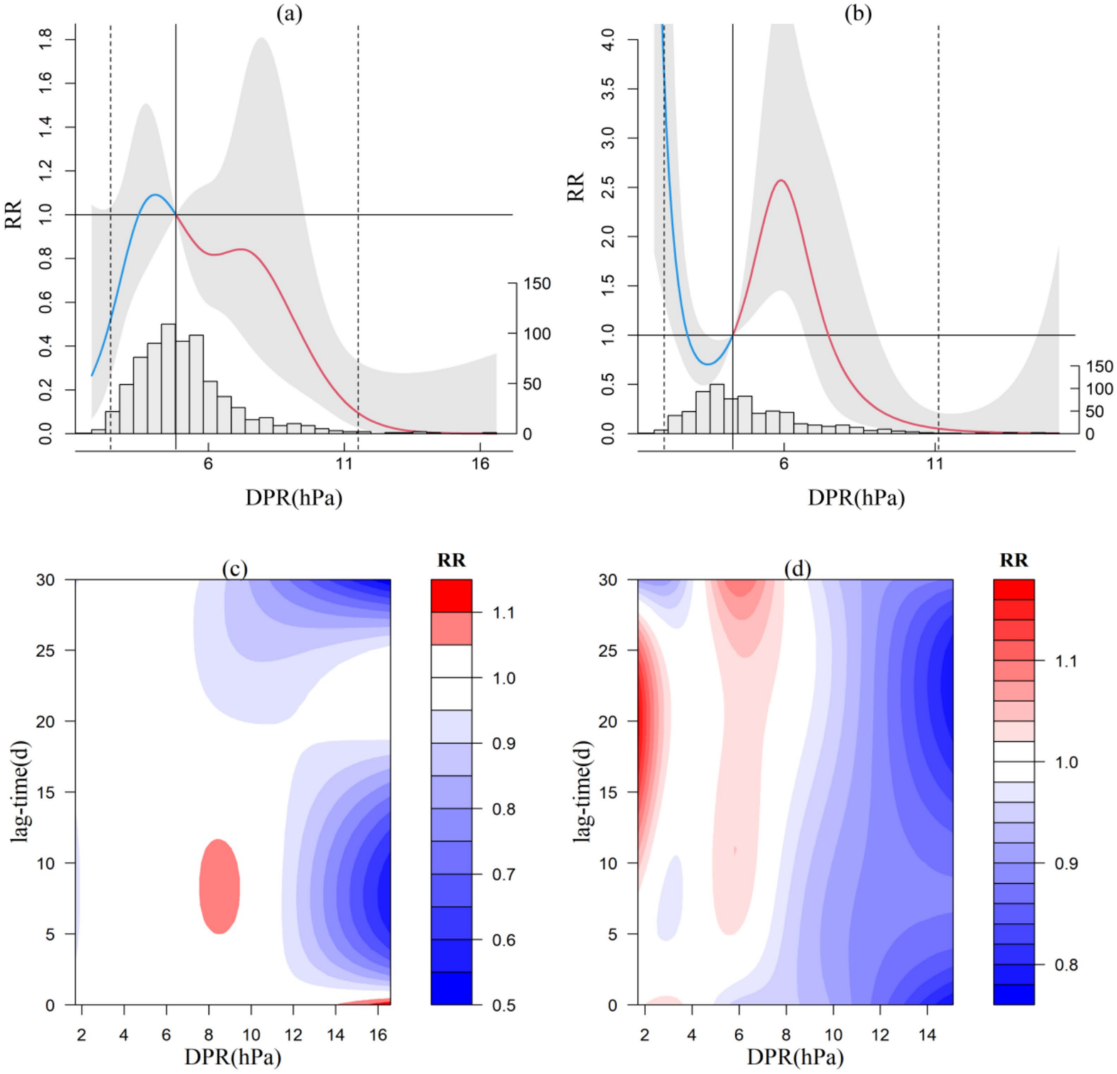
Exposure-response relationship between DPR and CVD incidence in Jiangkou and Xishui (a,b) and lag effect diagram (c,d; the gray area in a and b represents the 95% confidence interval for relative risk).

The change of barometric pressure also influenced the incidence of CVD. Setzer et al. (25) found that the increased incidence of aneurysm subarachnoid hemorrhage (aSAH) caused by changes in atmospheric pressure could be caused by the following factors: ① Intracranial pressure changes with changes in atmospheric pressure and the intracranial stress may increases. ② Systolic blood pressure may increase with the increase of atmospheric pressure, both of which are easy to induce aneurysm rupture. The exposure-response relationship between DPR and CVD incidence varies significantly across the four microclimate zones. In the Central Subtropical region (Jiangkou; Figure 3a) and the warm temperate region (Shuicheng, not shown), the relationship displays an irregular parabolic distribution. A slight risk of CVD incidence occurs when DPR is between 3.5–4.7 hPa or 3.2–4.3 hPa, with the highest RR values reaching 1.09 (95% CI: 0.82–1.45) and 1.15 (95% CI: 0.94–1.43), respectively. DPR in Jiangkou and ShuiCheng mainly play a protective role. Conversely, the impact of DPR is more pronounced in the South Subtropical region (Ceheng, not shown) and the North Subtropical region (Xishui), as depicted in Figure 3. In Ceheng, the RR follows a “W”-shaped distribution (not shown), and in Xishui it exhibits an inverted “N”-shaped distribution (Figure 3b). In Ceheng, significant risks of CVD incidence are noted when DPR is between 5.9 and 7 hPa. When the next day’s pressure falls below 3.1 hPa or exceeds 12 hPa, the RR increases linearly, with each 1 hPa change raising the RR by over 3.46. However, when DPR is between 3.2 ~ 5.9 hPa and 7.1 ~ 11.9 hPa, the RR is less than 1, and DPR may have a protective effect. In Xishui, DPR below 2.7 hPa increases the CVD incidence risk by 5.6 per 1 hPa decrease. Risks are considerable when the range is between 4.4 and 7.4 hPa, with the highest RR value reaching 2.57 (95% CI, 1.46–4.55). when DPR is between 2.8 and 4.3 hPa or higher than 7.5 hPa, the RR is less than 1, and DPR may have a protective effect.

Regarding the lag effect of DPR on CVD incidence, differences are noted among the representative stations in each climate zone. The DPR for ranges from 9 to 11 hPa in Jiangkou has a lag protection effect of 5 to 17 days, and a lag risk within 20 days above 13 hPa. The warm temperate zone (Shuicheng) mainly shows a delayed protection effect. In the South Subtropical representative station (Ceheng, not shown), a large DPR (12–16 hPa) shows a lag effect within 20 days. At the North Subtropical representative station (Xishui; Figure 3d), smaller DPR exhibits medium-to-long-term lag effects, with a lag of 8–27 days for ranges below 3 hPa and a lag of 5–30 days for ranges between 5 and 7 hPa, but DPR may have a protective effect within 30 days when it is higher than 8 hpa.

### 4.5 Pressure change between neighboring days

**Figure 4:**
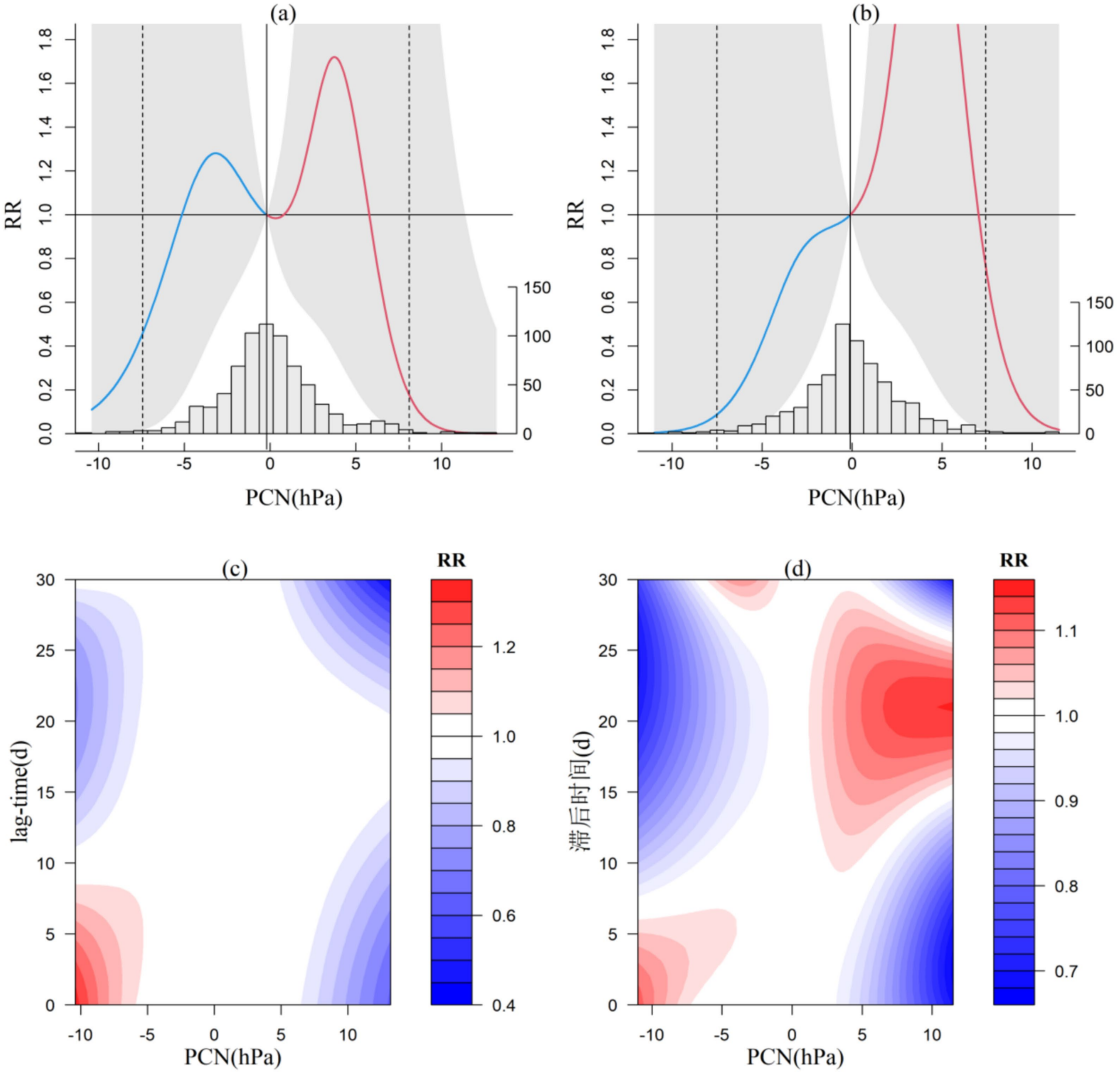
Exposure-response relationship between 24-h pressure change and CVD incidence in Jiangkou and Xishui (a,b) and lag effect diagram (c,d; the gray area in a and b represents the 95% confidence interval for relative risk).

This paper mainly analyzes Jiangkou (Figure 4a) and Xishui (Figure 4b) because PCN of Ceheng and Shuicheng is not so important to CVD in the decision tree model. In the Central Subtropical representative station (Jiangkou), an “M”-shaped distribution is noted, with an increased risk of CVD incidence when the pressure fluctuates between 0.9 and 5.7 hPa or falls below 5.1 hPa. When PCN rises by more than 5.7 hPa or drops by more than 5.1 hPa, it may exhibit a protective effect on CVD patients. In the North Subtropical representative station (Xishui) the relationship exhibits an irregular parabolic distribution. When PCN increase less than 7 hPa, CVD patients may be at risk of disease onset. However, when it exceeds 7 hPa or decreases, it may have a protective effect on CVD patients.

From the lag effect of PCN, at the central subtropical station, Jiangkou (Figure 4c), a 9-day lag is observed when the air pressure decreases by more than 5 hPa. The northern subtropical station, Xishui (Figure 4d), experiences a 6-day lag when the barometric pressure decreases above 5 hPa, and a 14–25 day lag effect when the barometric pressure increases above 2 hPa. Another common feature of the two stations is that when there is a significant decrease or increase in air pressure, the lag protection effect is also very pronounced.

### 4.6 Minimum temperature

**Figure 5:**
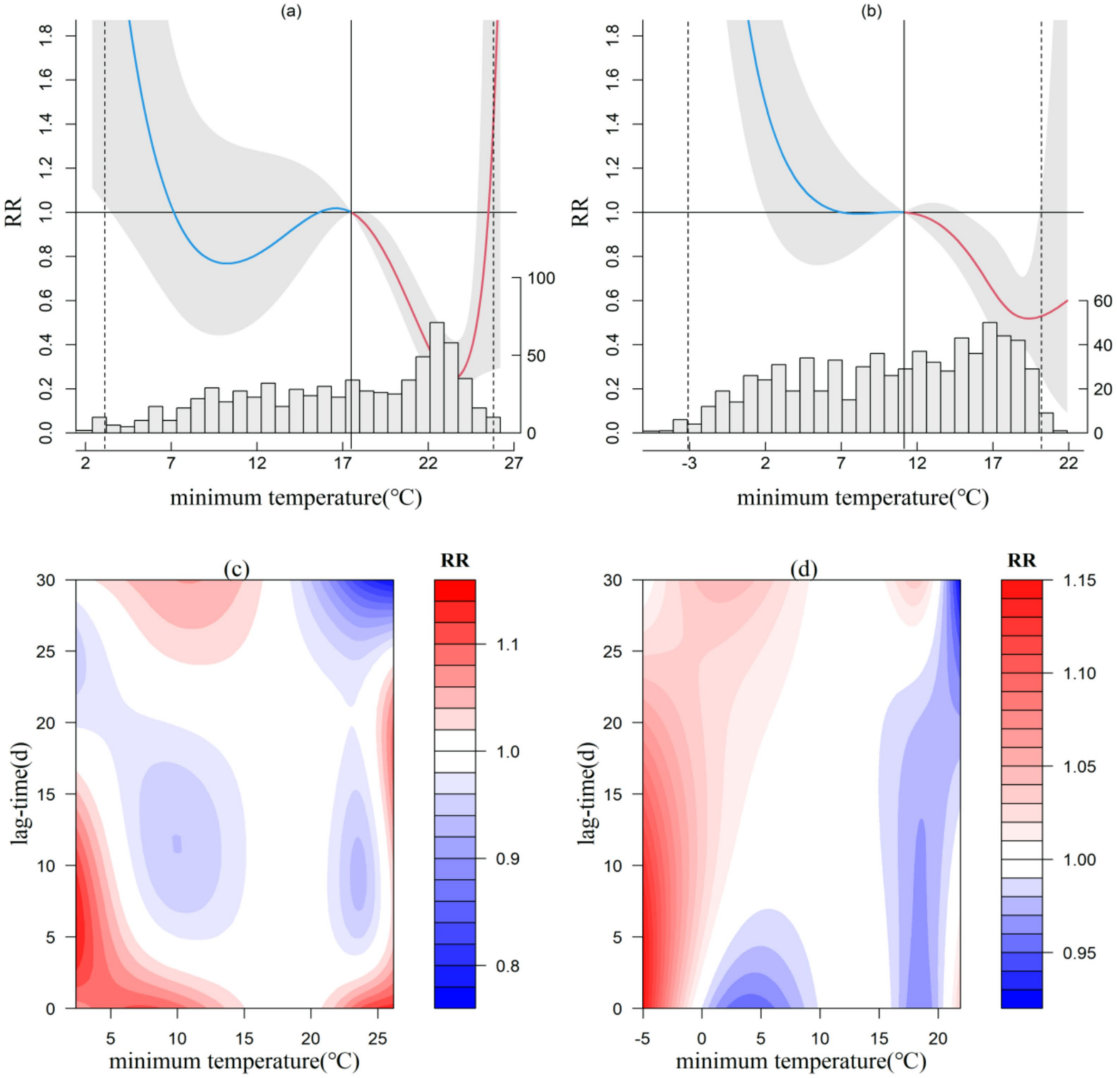
Exposure-response relationship between minimum temperature and CVD incidence in Ceheng and Shuicheng (a,b) and lag effect diagram (c,d; the gray area in a and b represents the 95% confidence interval for relative risk).

The exposure-response relationship between minimum temperature and CVD incidence is illustrated in Figure 5. In the South Subtropical region (Ceheng), the distribution follows a “W” shape, while the other three representative stations exhibit a near “ㄣ” shape. In Ceheng, when the minimum temperature is below 7.1°C or exceeds 25.6°C, the risk of CVD incidence increases almost linearly. For each 1°C decrease or increase in minimum temperature, the relative risk (RR) rises by 0.55 and 1.99, respectively. In the Central Subtropical, North Subtropical, and Warm Temperate regions, the risk of CVD incidence escalates when the minimum temperature drops below 12.2°C, 10.7°C, and 6.7°C, respectively. For each 1°C decrease in minimum temperature, the RR increases by 0.11, 0.18, and 1.04, respectively. Notably, in the warmer South Subtropical region, both low and high minimum temperatures significantly impact CVD incidence, whereas in the Central Subtropical, North Subtropical, and Warm Temperate regions, only low minimum temperatures are impactful. Additionally, the threshold for impact decreases with a reduction in the annual average temperature, suggesting a degree of climate adaptability among local populations.

Concerning the lag effect of minimum temperature on CVD incidence, lower minimum temperatures demonstrate a more pronounced lag effect, with varying impact thresholds across the microclimate zones. In the South Subtropical region (Ceheng), minimum temperatures below 15°C or above 21°C manifest short-term effects of 1–3 days, while temperatures below 5°C can extend the effect up to 15 days. In the Central Subtropical region (Jiangkou), temperatures ranging from 0–10°C and 20–25°C exhibit short-term effects of 1–2 days and long-term effects of 18–30 days. In the North Subtropical (Xishui) and warm temperate (Shuicheng) regions, very low temperatures (below 0°C) primarily show short-and medium-term effects, with a continuing lag effect of 3–18 days for Xishui and 0–27 days for Shuicheng.

## 5 Discussion

This paper delineates the common characteristics of the effect on the incidence of CVD of meteorological environments in various microclimate zones. Previous studies predominantly focused on meteorological factors within specific regions, which limited their applicability to local contexts. So this research is groundbreaking. This research underscores that the key sensitive meteorological factors identified include DTR, TCN, DPR, PCN and minimum temperature. There are no discernible pattern about the influence threshold and lag time of other meteorological environmental factors across the four representative microclimate stations. The results partially align with findings from other regions (19). In Bari, Southern Italy, significant meteorological factors for CVD incidence include average temperature, maximum temperature, perceived temperature, and relative humidity (26). In Tianjin, classified as a warm temperate region (27, 28), key meteorological factors influencing stroke encompass pressure, temperature, DTR, TCN, and notably correlated with DTR.

We compare and analyze the effects of temperature and its variability on CVD across four distinct microclimate zones, and discuss the adaptation of CVD patients to various climatic conditions. Echoing findings from Tianjin, CVD incidence across Guizhou’s diverse microclimate zones is strongly associated with temperature and its fluctuations (27). Common patterns observed involve a marked increase in CVD risk associated with a narrow DTR, a positive TCN and cold conditions (low minimum temperature). The influence of minimum temperature and TCN exhibit spatial heterogeneity. The thresholds of minimum temperature drops as the annual average temperature decreases. In the middle subtropical region of Jiangkou, the northern subtropical region of Xishui, and the warm temperate region of Shuicheng, there is a risk of CVD when the minimum temperature falls below 12.2°C, 10.7°C, and 6.7°C, respectively. The risk of CVD escalates with a positive TCN. In the four microclimate zones, the critical threshold for TCN causing CVD incidence also increases slightly with the local annual average temperature’s rising. When TCN exceeds the critical threshold (0.2°C in Shuicheng, 0.3°C in Xishui, and 0.4°C in Jiangkou and Ceheng), the risk of CVD incidence increases linearly. As with other studies, Extreme temperatures, either high (heatwaves) or low (cold spells), heighten the risk of CVD incidence or mortality in warm temperate areas such as Shijiazhuang (7), Jinan (29), and northern temperate regions like Pingyi, Shandong (30). In the central temperate region of Dingxi, Gansu (9), CVD hospitalization risks increase with temperatures dropping below or soaring above 0°C and when 24-h temperature changes are minimal (below 3°C). In the warm temperate region of Fuyang, Anhui, and the Central Subtropical region of Jinping, Guizhou (30), ischemic cardiovascular disease risks significantly rise with large DTR (15°C) and sharp 24-h temperature declines (over 7°C). In Ganzhou, Jiangxi, a Central Subtropical region (31), a positive correlation exists between the annual average temperature and maximum DTR.

In addition, there is no obvious relationship between the atmospheric pressure on CVD and the distribution of microclimate zone. DPR in middle subtropical region of Jiangkou and warm temperate region of Shuicheng may play a protective role, while the risk of the disease increases linearly when DPR is lower than 3.2 hPa and 2.7 hPa in south subtropical region of Ceheng and north subtropical region of Xishui, respectively. The effect of PCN on Ceheng and Shuicheng is not obvious. In Georgia (11) and Prague (12), reductions in atmospheric pressure correlate with increased stroke hospitalizations or CVD cases. Conversely, in Tianjin, CVD incidence shows a positive association with both average and minimum atmospheric pressure (27). Research from Kaunas (32) suggests that stroke risk escalates with daily mean atmospheric pressure fluctuations exceeding 3.9 hPa. Similar findings have been reported in Korea, where variations in DPR are significantly linked to ischemic strokes, with correlations varying by age group.

The advantage of this study is to find out the common meteorological environmental factors that affect the incidence of CVD in different microclimate zones in Guizhou mountainous areas. The results show that there is spatial heterogeneity in temperature and temperature variability, which provides a reference for climate adaptability decision-making in CVD prevention and control work in the future. Secondly, we analyze the threshold and lag time effects of temperature, air pressure and their variability on CVD in different microclimate zones, which is conducive to the targeted and accurate meteorological forecast and early warning in CVD prevention and control work. Some limitations in the present study should also be noted. The time of CVD cases in this study is 2021–2022, which is during the new coronavirus epidemic. There may be incomplete statistics of cases due to the epidemic containment controls, some bias cannot be avoided. In addition, we uses the monitoring values of meteorological elements of meteorological stations to evaluate their correlation with CVD, but does not use indoor meteorological environment to analyze and evaluate, which may also lead to some deviations.

## 6 Conclusion

The meteorological environment in low-latitude mountainous areas has a significant impact on CVD. The main meteorological factors are temperature, air pressure and their variability. The effects of minimum temperature and TCN show obvious spatial heterogeneity. It is recommended to strengthen the research and application of the full integration of meteorology and health, establish a meteorological risk prediction and early warning system for CVD prevention and control. At last we can provide correspongding protection measures for CVD individuals.

## Data Availability

The original contributions presented in the study are included in the article/supplementary material, further inquiries can be directed to the corresponding author.
